# Multilevel barriers to guideline implementation: a nationwide multi-professional cross-sectional study within child and adolescent psychiatry

**DOI:** 10.1186/s13034-024-00803-2

**Published:** 2024-09-12

**Authors:** Anna Helena Elisabeth Santesson, Robert Holmberg, Martin Bäckström, Peik Gustafsson, Sean Perrin, Håkan Jarbin

**Affiliations:** 1 Department of Clinical Sciences, Faculty of Medicine, Lund University, BMC F12, Lund, 221 84 Sweden; 2 Department of Psychology, Faculty of Social Sciences, Lund University, Box 213, Lund, 221 00 Sweden; 3 Child and Adolescent Psychiatry, Region Halland, Halland, 30185 Sweden

**Keywords:** Adolescents, Young people, Mental health, Depression, Guideline, Implementation, Barriers, Facilitators, Needs assessment, Multiprofessiona

## Abstract

**Background:**

Despite efforts to promote guideline use, guideline adoption is often suboptimal due to failure to identify and address relevant barriers. Barriers vary not only between guidelines but also between settings, intended users, and targeted patients. Multi-professional guidelines are often used in child and adolescent mental health services (CAMHS), making the implementation process more difficult. Despite this, there is a lack of knowledge about which barriers to consider or if barriers vary by profession. The aim of this study was to address these gaps by examining barriers to adopting a multi-professional depression guideline in the context of a nationwide implementation study.

**Methods:**

440 CAMHS clinicians across Sweden (52%) completed the Barriers and Facilitators Assessment Instrument (BFAI) ahead of an implementation endeavour. BFAI is a widely used and validated measure of guideline implementation on four scales: Innovation, Provider, Context, and Patient. Barriers were calculated at scale and at item levels. ANOVA and chi-square tests were used to analyse differences by profession and effect sizes were calculated.

**Results:**

Overall, clinicians were optimistic about guideline uptake, particularly about guideline characteristics and their own adoption ability. Barriers were related to the patient and the context domains, as well as to individual clinician knowledge and training. Perceptions differed across professions; psychiatrists were most, and counsellors were least positive about guideline embeddedness.

**Conclusion:**

This large-scale quantitative study suggests that CAMHS clinicians have an overall favourable attitude towards guideline adoption but highlights the need for adaptations to certain patient groups. Strategies to improve guideline use should primarily address these patient issues while securing proper support to the implementation. Implementation efforts, particularly those targeting staff knowledge, training, and involvement, may benefit from being tailored to different professional needs. These findings may inform implementation projects in CAMHS and future research.

**Supplementary Information:**

The online version contains supplementary material available at 10.1186/s13034-024-00803-2.

## Background

Clinical practice guidelines have the potential to improve the quality of care, but their uptake is slow and inconsistent due to multilevel barriers [1–3]. This also seems to apply to guidelines for youth depression in child and adolescent mental health services (CAMHS) [4, 5]. Compliance varies substantially across guideline recommendations, depression severity and patient groups [5, 6]. To implement new guidelines into routine practice, (CAMHS) clinicians often need to make substantial changes to their practice behaviour and are therefore particularly important stakeholders [3]. Yet, not much is known about which factors they perceive as most important nor if there are any differences due to professional background.

Youth depression is a critical and increasing public health problem with a range of negative outcomes and long-term effects, yet youths with depression still have significant unmet needs and are disadvantaged in receiving optimal care [7–9]. Evidence-based treatments exist but reach few and are often delivered in “sub-optimal” formats in routine care, and thus may be less effective compared to the outcomes achieved under research conditions [4, 6, 8, 10]. Clinical practice guidelines for depression aim to bring routine care practices more in line with the evidence base, particularly regarding assessment and assignment to, delivery of, and monitoring of evidence-based treatments [10–13].

Guidelines will only achieve their aims if they are used, and this will be largely (but not solely) dependent upon the attitudes of the individuals responsible for adopting and implementing the guidelines [14, 15]. The ability to adopt and sustain guideline use may be hampered by a range of factors some of which are related to the professional, such as awareness, training, and involvement [1, 2, 15, 16]. Clinicians’ beliefs about the clinical applicability and utility of guidelines, and how these relate to the individual needs of their particular patients, as well as the perceived complexity of the guidelines, and views about organisational supports for implementation are all likely to influence guideline adoption [1, 2, 17, 18]. Identification of guideline-related views may help in improving stakeholder involvement through more tailored adoption strategies [1, 17, 19]. For example, studies from (mental) health settings suggest that there may be differences in perceived barriers by profession; physicians seem to be more familiar with and ready to use guidelines [1, 2, 20–22]. However, little is known about potential enablers and obstacles to guideline implementation in CAMHS, including any variation between profession, about the applicability of care guidelines, and how they are best implemented. Currently, only a handful of studies have been carried out to examine CAMHS clinicians’ attitudes towards guidelines [5, 23–25]. In spite of depression being one of the most common condition treated in CAMHS, most studies on depression guideline adoption have been qualitative and retrospective, often involving small and non-representative samples, have not been able to study differences between professions, and take a pessimistic view [4, 5, 24]. There is a need for larger and more representative studies involving quantitative methods.

The Barriers and Facilitators Assessment Instrument (BFAI) is a theory-based measure that was developed to investigate modifiable barriers for implementation [26]. It measures individual barriers and facilitators as well as clusters of determinants in four domains: Innovation, Provider, Patient and Context. The BFAI is one of few instruments of guideline implementation that has been used in a range of implementation research projects including those targeting depression and mental health [17, 27–30].

Project Deplyftet is a nationwide implementation program that began in 2014 with the aim of improving the quality of care for depressed youth in Swedish CAMHS [31]. All publicly funded CAMHS were invited to participate, and implementation was carried out in 15 clinics across Sweden employing more than 1400 clinicians [32]. As part of that program, the BFAI was administered at the start of the implementation program.

The aim of the present study is to address a gap in the literature concerning CAMHS clinicians´ attitudes towards guideline implementation. Albeit important for patients, families, clinicians and society, evidence in implementing guidelines in CAMHS are scarce. This study will investigate CAMHS clinicians’ overall view on the feasibility of adopting a clinical guideline, which factors they perceive as most important and any professional differences. We report the scores on the BFAI, including its scales, and how these relate to professional status of the clinicians.

## Methods

### Design and setting

This cross-sectional study used a subsample of data drawn from clinicians working at 10 of 31 eligible CAMHS serving about 550 000 youth (26% of Swedish children) from 2016 to 2018 [32, 33]. The participating CAMHS represent all types of publicly owned and funded CAMHS serving similar-sized catchment areas (25,000−100,000) as the remaining CAMHS (i.e., average = 65,000 youth, range = 41,000−125,000).

All first-line clinicians in the participating CAMHS received to their work-e-mail a secure link to an electronic questionnaire. Up to five reminders were sent (if necessary).

### Participants/adopters

The sample consisted of 440 of 854 eligible clinicians resulting in a response rate of 52% (Supplemental Fig. 1 + Table S1). The typical participant was female (84%), had a bachelor’s degree (61%) and had five or less years in child and adolescent psychiatry (47%) (Table 1). For more detailed information on background characteristics per professional group, please see Supplemental Table 2 (S2).

**Table 1:** Background characteristics of respondents with at least one item response (*n* = 440)

		*n*	%
Gender			
	Male	69	15.9
	Female	364	84.1
Age group			
	< 35 years	90	20.8
	36–44 years	111	25.6
	45–55 years	117	27.0
	> 55 years	115	26.6
Education			
	Low	11	2.5
	Bachelor	291	67.1
	Master	115	26.5
	PhD	17	3.9
Profession			
	Auxiliary nurse	11	2.5
	Nurse	61	13.9
	Counsellor	121	27.6
	Psychologist	128	29.2
	Psychiatrist	57	13.0
	Other	60	13.7
Tenure child Mental health			
	< 5 years	207	47.9
	5–10 years	62	14.4
	11–15 years	51	11.8
	16–20 years	47	10.9
	> 20 years	65	15.0
	Don´t know	52	12.3

Sample sizes vary slightly due to missing data

*Psychiatrists include child psychiatrists, residents, and MDs without specialist training

### The innovation

The Swedish National Board of Health and Welfare (NBHW) published a depression and anxiety guideline 2010 aimed at decision-makers with the purpose to support politicians and healthcare managers at the regional level to identify evidence-based treatments that should be prioritised and to allocate adequate resources for delivering these treatments [34]. The Swedish Association for Child and Adolescent Psychiatry, an association under the Swedish Medical Association, developed a clinical practice guideline in 2014 based on the NBHW guideline [35]. This multidisciplinary guideline with a stepped-care approach, including check lists and recommendations to clinicians, has many similarities with other youth guidelines regarding the care pathway, assessment and treatment processes [11]. Compared to other guidelines, the Swedish guideline recommends brief psychosocial intervention as a first step for mild to but also to moderate depression [36]. The brief intervention corresponds to the brief psychosocial intervention delivered in the IMPACT -study [37].

### The implementation program

The implementation program “Deplyftet” was co-designed with clinicians, managers, academics, national authorities, and patient representatives, based on the Grol and Wensing Implementation of change model [17, 31]. This process model is often used in guideline implementation studies [5, 17]. It uses different feedback loops for formative evaluation during the implementation process, thus involving adopters and end-users in the implementation process.

### Measures

The web survey included questions about the respondent’s age, gender, and professional background, followed by the Barrier and Facilitator Assessment Instrument [26].

### The Barrier and Facilitator Assessment Instrument

The BFAI is a flexible instrument consisting of 27 items in two parts [26, 38]. Part 1(items 1–16) are intended for guideline implementation and part 2 (items 17–27) for preventive care. Items are rated on a Likert scale ranging from 1 *(fully disagree)* to 5 *(fully agree)*. Researchers are free to add items and to add an extra response option “Not applicable” (N/A). The BFAI consists of four scales: (1) *Innovation*, (2) *Provider*, (3) *Patient*, and (4) *Context.* A higher composite score indicates more barriers. To compute scale scores, positively worded items (##1–3 and #16) are reversed. Internal consistency for the scales ranged from Cronbach alpha (α) 0.63−0.68 but was not reported for the total scale [26, 38]. Nevertheless, a BFAI Total score has been used [29, 39]. Estimated time for completion is about 15 min.

The BFAI has been translated and adapted for use in Sweden [33]. The Swedish version of the BFAI (S-BFAI) used the items intended for guideline implementation and five items from the preventive care part, the 5-point Likert scale and the extra N/A response category. S-BFAI has been tested for basic psychometrics using the same benchmarks as the developers. Item qualities and internal consistency was adequate and on par with or slightly better than the original version: Innovation α = 0.74; Provider α = 0.73; Context α = 0.70; Patient α = 0.73 and BFAI total scale α = 0.85 [33]. The dimensionality of the Swedish version was supported by factor analyses, although the Context scale had some issues due to the intra- and inter-domain correlations. All items were kept as they all were increasing content validity.

### Data analysis

SPSS statistical software version 27 along with Mplus version 8 for Generalized Linear Modelling (GLM) [40, 41]. Prior to the analyses, we examined items and scales for missing values, outliers and normality, homogeneity of variance, linearity, and multicollinearity, for a detailed description please see supplemental material. The N/A response option was hereafter treated as missing. Continuous data are presented with means and standard deviations and categorical data with frequencies and percentages. We recoded some demographic variables. At the item level, according to the instructions for use, barriers were defined by collapsing response categories 1 and 2 for positively worded items and 4 and 5 for negatively worded items, facilitators were defined the opposite way around [38]. Scale scores were the means of test items, using the original 1–5 scale, provided at least 50% of scale items had valid data.

We used Generalized Linear Modelling (GLM) to investigate differences between scale means, Chi Square, and one-way ANOVA to test for between-group differences and correlation analyses to test the relationship between continuous and ordinal variables. We applied a Bonferroni correction to control for Type 1 error given multiple comparisons. To estimate the magnitude of the effect, we used Cramér’s V (V) and eta squared (η^2^). The following benchmarks were used: small V = 0.10, medium V = 0.30, large V = 0.50; small η^2^ = 0.01, medium η^2^ = 0.09, large η^2^ = 0.25 respectively [42, 43]. In the event of significant main effects in the ANOVAs, post-hoc comparisons were computed using Tukey HD tests. The reporting of results was guided by the Standards for reporting Implementation studies (STaRI) and the Strengthening the Reporting of Observational Studies in Epidemiology (STROBE) statements, respectively [44, 45].

## Results

### Clinician’s perceptions of barriers and facilitators toward guideline implementation

#### Domain level barriers and facilitators

Composite scores for the scales were all below a neutral score of 3 (a higher score indicate more barriers) (Table 2). The Patient scale had the highest score followed by the Context, Provider and lastly the Innovation. The Patient and Context scale scores differed from the Provider and Innovation scores, but the mean differences were small (Table 3).

**Table 2:** Descriptive statistics for scales and items of the Barrier and Facilitators Assessment Instrument

BFAI subscales and total and items within each subscale	Fully disagree %	Disagree %	Neutral %	Agree %	Fully agree %	*N*/A %	Miss %	M	SD	*N*
Innovation*								2.56	0.54	391
1. This guideline leaves enough room for me to make my own conclusion*	0.5	4.5	45.8	42.4	6.8	9.8	3.9	3.51	0.70	440
2. This guideline leaves enough room to weigh the wishes of the patient*	0.5	5.2	46.9	40.3	7.1	10.0	3.2	3.48	0.72	382
3. This guideline is a good starting point for self-study*	0.8	4.0	51.9	34.1	9.1	11.4	4.1	3.47	0.74	372
13. Working according to this guideline is too time consuming	7.9	34.2	45.1	10.9	1.9	13.4	3.4	2.65	0.85	366
14. This this guideline does not fit into my ways at working at my practice	8.5	39.1	42.0	8.5	1.9	11.1	3.4	2.56	0.84	376
16. The layout of this guideline makes it handy to use*	0.3	5.6	55.9	32.2	5.9	16.4	3.2	3.38	0.66	354
Provider								2.62	0.63	421
4. I did not truly read nor remember this guideline	16.5	26.7	32.8	14.3	9.7	3.9	2.5	2.74	1.18	412
5. I wish to know more about the guideline before I decide to apply it	7.0	16.2	15.2	38.4	23.2	1.8	1.1	3.55	1.20	427
6. I have problems changing my old routines	26.1	50.8	18.1	4.5	0.5	1.8	1.6	2.02	0.82	425
7. I thinks parts of the guideline are incorrect	13.7	31.9	47.7	5.6	1.1	12.0	2.5	2.49	0.84	373
8. I have a general resistance to working according to protocols	35.4	39.1	16.9	8.0	0.7	1.1	1.8	2.00	0.95	427
21. It is difficult to give care according to the guideline because I’m not trained	10.7	19.2	24.9	30.7	14.5	5.2	3.6	3.19	1.21	401
22. It is difficult to give care according to the Guideline because I’m not involved in setting it up	21.6	37.1	29.8	9.3	2.3	5.2	4.1	2.34	0.99	399
Context								2.64	0.64	356
9. Colleagues do not cooperate in applying the guideline	8.8	31.6	47.8	11.0	0.8	13.0	4.3	2.63	0.82	364
10. Other do not cooperate in applying the guideline	8.1	33.1	46.7	11.7	0.6	13.0	5.2	2.64	0.81	360
11. Managers do not cooperate in applying the guideline	15.3	35.8	38.0	10.1	0.8	11.8	5.0	2.45	0.90	366
15. Working according to this guideline require financial compensation	7.0	21.8	51.7	15.1	4.5	7.0	3.6	2.88	0.90	358
Patient								2.69	0.62	366
12. Patients do not cooperate in applying the guideline	9.7	32.0	53.4	4.6	0.3	14.8	5.7	2.54	0.74	350
23. It is difficult to give care according to the Guideline to patients with different cultural background	5.6	27.3	48.3	17.5	1.1	14.1	5.0	2.81	0.83	355
25. It is difficult to give care according to the guideline to patients with low socioeconomic status	7.8	36.6	42.9	10.5	2.2	12.7	5.2	2.63	0.86	361
26. It is difficult to give care according to the guideline to younger patients < 13 years	6.8	36.8	47.9	12.5	2.8	14.3	5.9	2.75	0.87	351
BFAI total								2.65	0.43	371

Note: Items scores could range from 1 to 5. N/A = Not applicable, Miss = missing. A higher mean score indicates more barriers except for positive formulated items (##1–3 + 16). Seven multivariate outliers were excluded. Scale scores are mean scores provided that 50% of scale items had valid data. To calculate scale scores for the Innovation subscale items ## 1–3 + 16 were reversed

**Table 3:** Pairwise comparison of subscales by generalized linear model

Subscale		Mean difference	STD error	*P* ^a^	CI for differences	
					Lower	Higher
Innovation	Provider	0.00	0.03	1.00	− 0.079	0.079
	Context	− 0.11*	0.04	0.02	− 0.213	− 0.013
	Patient	− 0.14^*^	0.03	< 0.001	− 0.225	− 0.048
Provider	Context	− 0.11*	0.04	0.04	− 0.222	0.003
	Patient	− 0.14*	0.03	< 0.001	− 0.225	− 0.048
Context	Patient	0.024	0.04	1.00	− 0.127	0.080

Note, *n* = 336, STD error = standard error

*The mean difference is significant at the 0.05 level

^a^Bonferroni Adjustments for multiple comparison

### Item level barriers and facilitators

At the item level, patient cultural background, and financial issues (from the context domain) were only the fifth and fourth most important barriers respectively (Table 4). Main individual barriers were on the level of the provider and related to knowledge, training, and involvement. The three most important facilitators concerned changing routines, working according to protocols and involvement in the implementation planning process. Furthermore, support from managers and flexibility of the guideline were the fourth and fifth most important facilitators, respectively.

**Table 4:** Top 5 barriers and facilitators at the item level

Item	Top 5 Barriers	(Fully) agree	
		n	%
5.	I wish to know more about the guideline before I decide to apply it. *(Provider knowledge and motivation)*	261	61.6
21.	It is difficult to give care according to the guideline because I am not trained in giving care according to the guideline *(Provider training)*	181	45.1
4.	I did not truly read nor remember this guideline. *(Provider involvement)*	98	24.0
15.	Working according to this guideline requires financial compensation. *(Context financial compensation)*	70	19.6
23.	It is difficult to give care according to the guideline to patients with different cultural background. *(Patient cultural background)*	66	18.6

Item	**Top 5 Facilitators**	(Fully) *dis*agree	
		n	%
6.	I have problems changing my old routines. *(Provider working style)*	327	76.9
8.	I have a general resistance to working according to protocols. *(Provider role perception)*	317	74.5
22.	It is difficult to give care according to the guideline because I’m not involved in setting it up. (*Provider involvement)*	234	58.6
11.	Managers do not cooperate in applying the guideline. *(Context managers support)*	186	51.1
1.	This guideline leaves enough room for me to make my own conclusion* *(Innovation flexibility)*	187	49.2

Note. Barriers were defined by collapsing the response category fully disagree with disagree for items ##1–3 + 16 and fully agree with agree for ##4–15, 21 − 13 and 25–26

Facilitators were defined the opposite way around

*Item 1 is reversed

Table 2 also presents means and standard deviations at single item level, a higher mean score indicates more barriers except for positive formulated items in the Innovation scale (##1–3 + 16). Top five barriers and facilitators were about the same, with a slight difference in their order.

### Differences in perceptions of barriers and facilitators by profession

The results for each scale presented by demographic groups and professions are depicted in Supplemental Table S5. In general, psychiatrists scored lower (perceived less barriers) than other professions, (see below). Gender, age, and years of experience were not found to have significant correlations with any of the scales (S6). Educational level had a weak and negative correlation (– 0.13) with the Provider scale.

### Domain level differences by profession

There were statistical differences by profession for BFAI Total and Innovation, Provider, and Patient scale (Table 5). The effect ranged from small (Innovation and Patient, η^2^ = 0.05) to medium (Provider, η^2^ = 0.10 and Total, η^2^ = 0.09). Psychiatrists perceived less barriers than other professions, except for patient domain where there were no significant differences between psychiatrists and psychologists.

**Table 5:** Means, standard deviations, and one-way ANOVA analyses of BFAI domains and total

BFAI subscales and total	All		Psychiatrist		Nurse		Social worker		Psychologist		Other*		F	df	*p*	η^2^
	*M*	*SD*	*M*	*SD*	*M*	*SD*	*M*	*SD*	*M*	*SD*	*M*	*SD*				
Innovation	2.57	0.52	2.34_a_	0.54	2.62_b_	0.47	2.67_b_	0.53	2.52_b_	0.53	2.61_b_	0.47	5.15	4,370	< 0.001	0.05
Provider	2.63	0.63	2.15_a_	0.63	2.78_b_	0.56	2.75_b_	0.59	2.61_b_	0.63	2.68_b_	0.57	10.7	4,405	< 0.001	0.10
Context	2.64	0.64	2.51_a_	0.64	2.61_a_	54	2.68_a_	0.66	2.58_a_	0.69	2.82_a_	0.59	2.01	4,351	0.09	0.02
Patient	2.69	0.63	2.38_a_	0.63	2.76_b_	0.55	2.87_b_	0.62	2.64_ab_	0.62	2.73_b_	0.64	5.09	4,349	< 0.001	0.06
Total barrier	2.65	0.43	2.36_a_	0.42	2.68_b_	0.42	2.75_b_	0.39	2.63_b_	0.43	2.71_b_	0.38	8.39	4,364	< 0.001	0.09

* included auxiliary nurses. Note: Seven multivariate outliers were excluded. Scale scores are mean scores provided that 50% of scale items had valid data. Means not sharing subscripts differ significantly at α < 0.05 as indicated by Tukey´s HD

### Item level differences by profession

There were significant differences between perceived barriers at the item level in the provider domain by profession but not regarding financial issues and patient cultural background (Table 6). Psychiatrists differed from the other professions on item #5 “wish to know more” by being less likely to perceive it as a barrier. Counsellors perceived more training barriers compared to psychiatrists and psychologists. Finally, counsellors and psychologists more often “did not thoroughly read nor remember the guideline” compared with psychiatrists. However, the effect sizes were small (V ranging from 0.17 to 0.29).

**Table 6:** Top five barriers at the item level and difference by profession

BFAI item	All		Psychiatrist		Nurse		Counsellor		Psychologist		Other*		Χ^2^ (4)	*p*	V
	n	%	n	%	n	%	n	%	n	%	n	%			
5. I wish to know more about the guideline before I decide to apply it	261	61.4	16_a_	28.1	46_b_	76.7	71_b_	60.2	80_b_	65.0	48_b_	71.6	36.35	< 0.001	0.29
21. It is difficult to give care according to the guideline because I am not trained in giving	181	45.3	17_a_	30.4	26_ab_	47.3	61_b_	57.0	43_a_	37.4	34_b_	50.7	14.76	0.005	0.19
4. I did not truly read nor remember this guideline	98	23.9	4_a_	7.3	15_ab_	25.9	34_b_	30.9	31_b_	25.8	14_ab_	20.9	12.03	0.02	0.17
15. Working according to this guideline requires financial compensation	70	19.6	8_a_	15.1	15_a_	28.3	15_a_	16.5	20_a_	19.4	12_a_	21.1	3.87	0.42	0.10
23. It is difficult to give care according to the guideline to patients with different cultural background	66	18.6	6_a_	11.1	13_a_	25.5	16_a_	17.6	18_a_	18.0	13_a_	22.5	4.23	0.38	0.11

* included auxiliary nurses. Note. V = Cramér’s V. Barriers were defined by collapsing the response category fully disagree with disagree for items # #1–3 + 16 and fully agree with agree for items ##4–15, 21 − 13 and 25–26. A binary choice design was chosen; barriers were compared to non–barriers (neutral plus facilitators). Two participants did not report their profession. No cells have an expected count less than 5. Frequencies not sharing subscripts differ significantly at α < 0.05

Psychiatrists were more positive about guideline flexibility compared with the other professions and were more involved in the implementation planning process compared to nurses and counsellors (Table 7). Again, the effect sizes were small (V = 0.25 and 0.19 respectively). The other three top facilitators did not differ by profession.

**Table 7:** Top five facilitators at the item level and difference by profession

	All		Psychiatrist		Nurse		Counsellor		Psychologist		Other*		χ^2^(4)	*P*	V
	n	%	n	%	n	%	n	%	n	%	n	%			
6. I have problems changing my old routines	327	77.3	40_a_	71.4	44_a_	74.6	91_a_	78.4	93_a_	75.6	59_a_	85.5	4.29	0.369	0.10
8. I have a general resistance to working according to protocols	317	74.6	46_a_	80.7	42_a_	70.0	80_a_	69.6	95_a_	76.0	54_a,_	79.4	4.29	0.369	0.10
22. It is difficult to give care according to the guideline because I’m not involved in setting it up	234	58.9	41_a_	74.5	25_b_	45.5	54_b_	50.9	76_ab_	66.1	38_ab_	57.6	14.95	0.005	0.19
11. Managers do not cooperate in applying the guideline	186	51.1	35_a_	62.5	29_a_	55.8	44_a_	47.3	56_a_	52.8	22_a_	38.6	7.59	0.011	0.14
1.This guideline leaves enough room for me to make my own conclusion	187	49.5	43_a_	78.2	22_b_	38.6	41_b_	42.3	55_b_	50.5	26_b_	43.3	23.79	0.001	0.25

* included auxiliary nurses. Note. V = Cramér’s V. Facilitators were defined by collapsing the response category “fully agree” with “agree” for items ##1–3 + 16 and “fully disagree” and “disagree” for items #4–15, 21–23 and 25–26. A binary choice design was chosen; facilitators were compared to non-facilitators (barrier plus neutral). Two participants did not report their profession. No cells had an expected count less than 5

Frequencies not sharing subscripts differ significantly at α < 0.05

## Discussion

This is the first large-scale study examining barriers and facilitators to the implementation of a clinical practice guideline (depression) in a nation-wide sample of child and adolescent mental health clinicians. CAMHS clinicians were overall positive about adopting the depression guideline. At the domain level, they perceived fewer barriers regarding the characteristics of the guideline and their own ability to adopt the guideline, but more barriers regarding the context and patient characteristics. These findings suggest that CAMHS clinicians, despite carrying a positive attitude towards the guideline itself and the implementation, have concerns about support and clinical utility of the guideline for certain subgroups of patients. At the individual determinant level, the main facilitators to adoption concerned the clinician’s own role perception and working style, while lack of knowledge and training were main barriers, all in the provider domain.

Psychiatrists were in general more positive compared to other professions, and most so compared to counsellors. Psychiatrist perceived less barriers and more facilitators regarding their own capability to adopt the guideline, particularly concerning guideline awareness, training needs, and involvement.

### Clinician’s perceptions of barriers and facilitators toward guideline implementation

#### Overall view on the feasibility of guideline implementation

Overall, participants were positive to the guideline and its adoption adding to and at odds with previous qualitative studies on depression guideline implementation in CAMHS and youth mental health, mostly finding barriers [5, 24, 25]. Previous guideline studies using the BFAI have suggested a link between the overall perception of barriers and guideline use and adherence, a lower general barrier score was associated with higher use and better compliance [29, 46].

### Main barriers and facilitators at the domain level

A challenge in guideline implementation is concerns about guideline usefulness and ability to account for real world complexity [47]. It is problematic since perceived utility also seems to be linked to use in the context of CAMHS [23, 24]. In our study, clinicians held a positive view of the guideline as opposed to the results of the Westerlund study in which the NBHW guideline was perceived considerably less helpful, possibly because it was developed externally, not connected to the child mental health professional community, and did not take clinical expertise into account [24]. Successful implementation may be enhanced when guidelines are produced by experts in collaboration with the adopters with more of a bottom-up rather than a top-down approach [1]. Other possible explanations are differences regarding the innovation studied (clinical practice guideline versus guideline), their scope (depression among children, versus anxiety and depression across all ages), whom it was aimed at (clinicians versus decision makers) and content (stepwise recommendations how to care for the patients and checklist versus recommendation on how to prioritize between interventions) [24].

Guidelines are often criticised for focusing on the” average patient” and for not addressing the needs of vulnerable patient groups [1, 2, 48]. Although mainly positive, clinicians in our study held somewhat less positive attitude related to the patient domain, i.e. the difficulty of using the guideline with certain patient groups. This is in keeping with the studies of Westerlund et al. and Hetrick et al. where the most prominent barriers identified were at the patient level and the perceived fit between the guideline and the patients [24, 25]. However, the study of Hermens et al. found barriers at the context and provider level and not so much regarding the guideline itself or the patients [5].

### Main barriers and facilitators at the individual determinant level

Main individual barriers concerned provider knowledge, involvement, and training, in line with previous studies [5, 24, 25]. Familiarity with and intention to adopt the guideline were generally low in the Westerlund study [24]. Lack of training and availability of capable professionals were among the main barriers in the studies of Hermens and Hetrick [5, 25]. Our key facilitators were attitudes to follow guidelines and protocols, working style, and involvement in the implementation planning process. These aspects were not mentioned in the previous studies, but may be fundamental when designing strategies to enhance guideline adoption [2].

### Differences in perceptions of barriers and facilitators by profession

Not much is known about professional differences in attitudes toward guideline uptake in CAMHS. Previous studies suggest a relatively high concordance despite staff mix and different philosophies but did not investigate differences due to profession as such [24, 25]. We found important differences in the general view, across all except the context domain, and regarding half of the top barriers and facilitators. Overall, psychiatrists were more positive about the possibility of implementing the guideline and they were notably more positive about their own ability to adopt the guideline. A more fine-grained analysis showed that psychiatrists perceived less awareness, training, and involvement barriers than counsellors, but also compared to the other groups. Furthermore, psychiatrists perceived less patient domain barriers compared to counsellors, nurses and others but not compared to psychologists. However, regarding patient cultural background barrier, the perception did not differ across professions.

Psychiatrists were also more positive regarding characteristics of the guideline, although the magnitude was small. Notably, psychiatrists perceived the guideline as more flexible than the others, giving them more room to make their own conclusions. There are no comparable studies in CAMHS, but results are in line with a study in adult mental health, which found that psychiatrists held more favourable attitudes and less knowledge related barriers compared to the other professional groups [21]. A possible explanation for psychiatrists overall more positive attitudes may relate to the guideline being produced by the medical professional association, perhaps leading to a sense of ownership [46]. In addition, psychiatrists had been informed about the guideline during the development and were early on invited to comment drafts and hence may have reached another stage in the implementation process [1, 21].

#### Implications for guideline implementation projects

An important aspect of determination frameworks is that different barriers imply different types of measures and play a role for guideline development and stakeholder involvement. Our key findings expand on the previous results from youth depression implementation studies, which have found generally negative attitudes among clinicians towards implementation. In this study, CAMHS clinicians held an overall positive attitude toward guideline uptake, particularly about the characteristics of the guideline. However, they were somewhat less positive about patient factors, implying a need for information about how guideline recommendations might be adapted to vulnerable patients. The results at the individual determinant level also stress the importance of clear information about core components and to offer adequate education and training to improve guideline adoption. For guidelines to be implemented it may be necessary to clarify possible differences among professions [1]. This nationwide study allowed us to make these comparisons. While a consensus was observed about the context, there were differences in perceptions about important barriers across all other domains, notably regarding the provider. The results suggest that implementation efforts might benefit from being customized to the different needs of professional groups. To enhance guideline adoption, implementation participation and ownership issues should be addressed. Collaboration with more skeptical provider groups and inclusion of their perspective can inform the design, selection, and implementation of strategies to enhance the uptake of the guideline.

### Implications for research

More research is needed to investigate whether the barriers at the domain and determinants levels found in our and previous studies are so-called core determinants common to other guideline implementation studies in CAMHS or whether they are unique to youth depression guideline studies in general or specific to this study [49]. Further research should study CAMHS clinicians overall perception of the possibility of implementation and carefully distinguish which barriers are important for different professional groups and evaluate the effect of perceived barriers on the uptake of the guideline in daily practice A promising area for further research is to evaluate various tailored approaches to dissemination and implementation to calibrate interventions and develop cost effective and sustainable approaches. These and previous results also point to the need for inclusion of more vulnerable patients in treatment studies informing adolescent depression guidelines [4, 24, 25].

#### Strengths and limitations

The present study benefitted from the use of a standardised and validated self-report measure of views on implementation in a large sample of front-line CAMHS clinicians representing various professions. The participants were recruited from a geographically diverse area covering a third of Sweden’s CAMHS and representing a third of the Swedish regions (counties), with the sample’s characteristics being similar to available national data describing the CAMHS workforce [50]. The response rate of slightly over 50% is commonly viewed as sufficient for e-mailed surveys, and the sample size sufficiently large for the number and type of statistical analyses that were carried out.

A limitation was lack of descriptive data for non-respondents to investigate any potential selection bias, such that barriers could be over- or underestimated. The missing data pattern was not completely at random, which can introduce uncertainty and may reduce generalizability [42]. However, we did not find any differences between participants with complete versus incomplete data in demographic or profession. Reflecting the nature of the CAMHS workforce in Sweden, the professional groups differed in size [50]. Generally, professions with a more negative view outnumbered the more positive ones resulting in a more negative view for the entire sample. Finally, our findings are based entirely on a self-report measure. While validated in previous studies, we may have obtained different findings with another self-report measure and/or interviews. In any study of this kind, the influence of social desirability on responses cannot be excluded. In our case it might have reduced the scores for some of the facilitators in the Provider scale. Although previous studies have found a link between guideline attitudes and (non-) adherence, attitudes in this study may not reflect actual behaviour. Therefore, an audit is underway to investigate compliance with the guideline [51]. Finally, while the Swedish version has preliminary support for its dimensionality and reliability, the psychometric properties of the scales need further examination. The Context scale is brief, consisting of three homogeneous items. The fourth item, “Requires financial compensation”, may be an outlier but adds to content validity and was rated as a top barrier. Another limitation is the use of a sum score for all BFAI items. It is unclear whether the BFAI Total should be viewed as a scale with theoretically correlated variables (reflective indicators) tapping a common trait (latent variable), as an index composed of a list of variables (formative indicators), or as a combination of both, i.e., an index of four scales.

## Conclusions

Guidelines, if adopted, are likely to be important to the delivery (and adherence to) evidence-based treatments in CAMHS, but their adoption may be hampered by multilevel barriers. Our results suggest an overall positive attitude toward the adoption of this depression guideline in CAMHS in Sweden. Nevertheless, there are issues that need to be addressed to enhance guideline adoption and achieve an equitable care, namely the possibility of adaptation of guideline components to meet the needs of specific patient groups and the need for staff education and training. In addition, our results suggests that strategies for enhancing guideline use should be somewhat tailored by profession.

.

## Electronic supplementary material

Supplementary Material 1

## Data Availability

No datasets were generated or analysed during the current study.

## Abbreviations

ANOVA: Analysis of variance
BFAI: Barriers and facilitators assessment instrument
CAMHS: Child and adolescent mental health services
GLM: Generalized Linear Modelling
N/A: Not applicable
NBHW: Swedish National Board of Health and Welfare
S-BFAI: The Swedish version of the Barriers and facilitators assessment instrument
StaRI: Standards of Reporting Implementation Studies
STROBE: The Strengthening the Reporting of Observational Studies in Epidemiology
