# Supplementary material for: Multilevel barriers to guideline implementation: a nationwide multi-professional cross-sectional study within child and adolescent psychiatry
Source: Child Adolesc Psychiatry Ment Health. 2024 Sep 12;18:115. doi: 10.1186/s13034-024-00803-2 (PMC11397028; doi:10.1186/s13034-024-00803-2)
Supplement: Supplementary file 1 — Supplementary Material 1 [file 13034_2024_803_MOESM1_ESM.docx]

Supplemental material

Data analysis

SPSS statistical software version 27 was used for the analysis. Prior to the analyses, we examined the items and scales for missing values (supplemental tables S1-S3). Response was available for at least one item for 440 (96.7%) of 455 participants (S1). The non -applicable response option were hereafter treated as missing, and a missing value analysis was carried out. The missing pattern was not completely at random. There were no differences between the complete and incomplete samples due to demographical variables (S2). Basic statistics and normality tests (by examining histograms, skewness, kurtosis an q-q-plots) were then calculated (S4).

We used Generalized linear modelling (GLM) to investigate differences between scale means with a Bonferroni correction to control for type 1 error given multiple comparisons. Chi square-test was used to analyse potential differences at the item level by profession. A binary choice design was chosen; barriers were compared to non – barriers (neutral plus facilitators and facilitators were compared to non-facilitators (barrier plus neutral). We used Cramér’s to measure the strength of the association and standardized residuals to identify which groups were different to the others.

Correlation analyses were used to test the relationship between continuous and ordinal variables and several ANOVA tests to analyse potential differences on the scale level by profession. Homogeneity of variance was tested using Leven´s test and Hartley’s F _max_ in conjunction with sample size ratios using the benchmarks suggest by Tabanick and Fidell. We applied a Bonferroni correction (α=.05/4) to control for type 1 error given multiple comparisons. To estimate the magnitude of the effect, we used eta squared (η^2^) with the following benchmarks: small η^2^= .01, medium η^2^= .09, large η^2^= .25. In the event of significant effects, post-hoc comparisons were computed using Tukey HD tests.

Data preparation

Some demographic variables were recoded. We collected continuous data for age and tenure for the 2018 survey (CAMHS 6-10 in Supplemental table S1). These data were transformed to correspond with age and tenure categories for the 2016 survey. We also collapsed categories with small numbers after reviewing their frequency distributions; nurse assistants and others were collapsed to a new” others” category.

According to instructions for use, barriers at the item level was defined by collapsing the response category “Disagree” with “Fully disagree” (score >3 for positively worded items (#1-3+ #16) and “Disagree” with “Fully disagree” (score<3) for negatively worded items (##4-15+ 17-27) (Peters). Facilitators at the item level were defined the opposite way around.

Scale scores were the means of test items, provided at least 50% of scale items had valid data (n= 366 to 421). Seven multivariate outliers (Mahalanobis distance with p <0.001) were excluded. In addition, four within cell outliers were excluded from the Innovation scale, two from the Patient scale and one from the BFAI Total scale. A few cases were excluded due to missing data on profession, leaving 375 (Innovation), 410 (Provider), 356 (Context), 354 (Patient) and 366 (BFAI Total) cases for the one-way ANOVA analyses. For the scale comparisons a listwise deletion was used leaving 336 cases for the GLM.

All scales were reasonably normally distributed (supplemental table S4). Normal distribution was also tested for within groups of profession. Results of evaluation of assumptions of normality, homogeneity of variance, linearity, and multicollinearity for the scale and within groups were satisfactory.

Figures and tables

Figure 1 Study flow chart

| **Supplemental table S1** Response rate and BFAI item completion by sites | | | | | | |
| --- | --- | --- | --- | --- | --- | --- |
| CAMHS | Response  (n) | Approached  (n) | Response rate | Valid data for all BFAI items^a^ | Valid data in at least one BFAI item^a^ | Completion rate |
|  | n | n | % | n | n | % |
|  |  |  |  |  |  |  |
| 1 | 48 | 82 | 58 | 39 | 47 | 83 |
| 2 | 48 | 90 | 53 | 42 | 47 | 89 |
| 3 | 19 | 28 | 68 | 16 | 19 | 84 |
| 4 | 45 | 93 | 48 | 35 | 42 | 83 |
| 5 | 53 | 88 | 60 | 46 | 52 | 88 |
| 6 | 65 | 149 | 45 | 21 | 62 | 34 |
| 7 | 24 | 41 | 59 | 10 | 24 | 42 |
| 8 | 66 | 125 | 53 | 23 | 63 | 36 |
| 9 | 47 | 85 | 55 | 19 | 46 | 41 |
| 10 | 40 | 73 | 55 | 17 | 38 | 45 |
| All | 455 | 854 | 53 | 268 | 440 | 61 |
| ^a^The ”not applicable” response option is treated as missing | | | | | | |

| **Supplemental table S2** Descriptive characteristics of study participants by profession | | | | | | | | | | | | | |
| --- | --- | --- | --- | --- | --- | --- | --- | --- | --- | --- | --- | --- | --- |
|  | | | | | | | | | | | | | |
|  |  | Total  (n=440) | | Nurse  (n=61) | | Social  Worker  (n=121) | | Psychologist  (n=128) | | Psychiatrist  (n=57) | | Other  (n=71) | |
|  |  | n | % | n | % | n | % | n | % | n | % | n | % |
| Gender |  |  |  |  |  |  |  |  |  |  |  |  |  |
|  | M | 69 | 15.9 | 5 | 8.2 | 12 | 10.1 | 26 | 20.6 | 14 | 25.0 | 12 | 6.9 |
|  | F | 363 | 84.1 | 56 | 91.8 | 107 | 89.9 | 100 | 79.4 | 42 | 75.0 | 59 | 83.0 |
|  | missing | 7 | 1.6 | 0 | 0 | 2 | 1.6 | 2 | 1.6 | 1 | 1.8 | 0 | 0 |
| Age Group |  |  |  |  |  |  |  |  |  |  |  |  |  |
|  | <35 years | 90 | 20.8 | 7 | 11.5 | 19 | 15.8 | 42 | 33.3 | 13 | 23.2 | 9 | 12.9 |
|  | 35-44 years | 111 | 25.6 | 17 | 27.9 | 32 | 26.7 | 39 | 31.0 | 11 | 19.6 | 12 | 17.1 |
|  | 45-55 years | 117 | 27.0 | 18 | 29.5 | 41 | 34.2 | 25 | 19.8 | 13 | 23.2 | 20 | 28.6 |
|  | >55 years | 115 | 26.6 | 19 | 31.1 | 28 | 32.3 | 20 | 15.9 | 19 | 33.9 | 29 | 41.4 |
|  | Missing | 7 | 1.6 | 0 | 0 | 1 | .8 | 2 | 1.6 | 1 | 1.8 | 1 | 1.4 |
|  |  |  |  |  |  |  |  |  |  |  |  |  |  |
| Education |  |  |  |  |  |  |  |  |  |  |  |  |  |
|  | Low | 11 | 2.5 | 0 | 0 | 1 | .8 | 0 | 0 | 0 | 0 | 10 | 14.3 |
|  | Bach | 291 | 67.1 | 50 | 82.0 | 99 | 81.8 | 58 | 45.7 | 38 | 69.1 | 46 | 65.7 |
|  | Ms | 115 | 26.5 | 11 | 18.0 | 21 | 17.4 | 64 | 50.4 | 5 | 9.1 | 14 | 20.0 |
|  | PhD | 17 | 3.9 | 0 | 0 | 0 | 0 | 5 | 3.9 | 12 | 21.8 | 0 | 0 |
|  | Missing | 6 | 1.4 | 0 | 0 | 0 | 0 | 1 | .8 | 2 | 3.5 | 1 | 1.4 |
|  |  |  |  |  |  |  |  |  |  |  |  |  |  |
| Tenure Child  Mental health |  |  |  |  |  |  |  |  |  |  |  |  |  |
|  | <5 years | 207 | 47.9 | 25 | 41.7 | 69 | 57.0 | 66 | 52.4 | 15 | 26.8 | 32 | 46.4 |
|  | 5-10 years | 62 | 14.4 | 11 | 18.3 | 17 | 14.0 | 19 | 15.1 | 13 | 23.2 | 2 | 2.9 |
|  | 11-15 years | 51 | 11.8 | 11 | 18.3 | 11 | 9.1 | 17 | 13.5 | 7 | 12.5 | 5 | 7.2 |
|  | 16-20 years | 47 | 10.9 | 3 | 5.0 | 17 | 14.0 | 9 | 7.1 | 9 | 16.1 | 9 | 13.0 |
|  | >20 years | 65 | 15.0 | 10 | 16.7 | 7 | 5.8 | 15 | 11.9 | 12 | 21.4 | 21 | 30.4 |
|  | Missing | 8 | 1.8 | 1 | 1.6 | 0 | 0 | 2 | 1.6 | 1 | 1,8 | 2 | 3.3 |
| *Psychiatrists include child psychiatrists, residents and MDs without any specialist training, others include nurse assistants and others, | | | | | | | | | | | | | |

| Supplemental table S3 Missing value analysis by demographic characteristics | | | | | |
| --- | --- | --- | --- | --- | --- |
|  | Valid data for all BFAI items^a^  N=268 | | Missing data in at least one item^b^  N=187 | | *p* |
|  | n | % | n | % |  |
| Gender |  |  |  |  | .66 |
| Male | 43 | 16.3 | 27 | 14.8 |  |
| Female | 221 | 83.7 | 156 | 85.2 |  |
| missing | 4 | 1.5 | 4 | 2.1 |  |
| Age Group |  |  |  |  | .23 |
| <35 years | 62 | 23.5 | 33 | 18.0 |  |
| 35-44 years | 63 | 23.9 | 50 | 27.3 |  |
| 45-55 years | 76 | 28.8 | 45 | 24.6 |  |
| >55 years | 63 | 23.9 | 55 | 30.1 |  |
| Missing | 4 | 1.5 | 4 | 2.1 |  |
|  |  |  |  |  |  |
| Education |  |  |  |  | .46 |
| Low | 8 | 3.0 | 4 | 2.2 |  |
| Bachelor | 174 | 65.4 | 126 | 69.2 |  |
| Mater | 76 | 28.6 | 43 | 23.6 |  |
| PhD | 8 | 3.0 | 9 | 4.9 |  |
| Missing | 2 | .7 | 5 | 2.7 |  |
|  |  |  |  |  |  |
| Profession |  |  |  |  | .95 |
| Auxiliary nurse | 7 | 2.6 | 5 | 2.7 |  |
| Nurse | 40 | 17.6 | 27 | 14.6 |  |
| Counsellor | 72 | 27.0 | 51 | 27.6 |  |
| Psychologist | 75 | 28.1 | 57 | 30.8 |  |
| Psychiatrist | 37 | 13.9 | 20 | 10.8 |  |
| Other | 36 | 13.5 | 25 | 13.5 |  |
| Missing | 1 | .4 | 2 | 1.1 |  |
| Tenure Child  Mental health |  |  |  |  | .23 |
| <5 years | 135 | 51.1 | 82 | 45.1 |  |
| 5-10 years | 37 | 14.0 | 25 | 13.7 |  |
| 11-15 years | 33 | 12.5 | 18 | 9.9 |  |
| 16-20 years | 22 | 8.3 | 26 | 14.3 |  |
| >20 years | 27 | 14.0 | 31 | 17.0 |  |
| Missing | 4 | 1.5 | 5 | 2.7 |  |
| Note The non applicable response option is treated as missing., *p= p*-value. The p-value is based on the Pearson Chi-square.  *Psychiatrists include child psychiatrists, residents and MDs without any specialist training | | | | | |

| **Supplemental table 4** Central tendency, dispersion, and distribution for scales in the different samples | | | | | | | | | | | |
| --- | --- | --- | --- | --- | --- | --- | --- | --- | --- | --- | --- |
|  |  | N | M | SD | Skewness | Kurtosis | Median | Min | Max | Range | IQ-range |
|  |  |  |  |  |  |  |  |  |  |  |  |
| Innovation | All ^a^ | 407 | 2.55 | .54 | 0.12 | .65 | 2.67 | 1.00 | 5.00 | 4 | .83 |
|  | 50 %^b^ | 391 | 2.56 | .53 | -0.10 | -0.61 | 2.67 | 1.17 | 5.00 | 3.83 | .83 |
|  | 50% minus outliers^c^ | 382 | 2.57 | .52 | -0.12 | -0.66 | 2.67 | 1.17 | 5.00 | 3.83 | .83 |
|  | No missing in scale^d^ | 322 | 2.57 | .49 | -0.37 | -0.38 | 2.67 | 1.20 | 3.83 | 2.67 | .83 |
|  | No Missing sample^e^ | 268 | 2.57 | .41 | -0.37 | -0.38 | 2.67 | 1.33 | 3.83 | 2.67 | .84 |
|  |  |  |  |  |  |  |  |  |  |  |  |
| Provider | All ^a^ | 440 | 2.65 | .67 | -0.04 | 0.07 | 2.71 | 1.00 | 5.00 | 4 | .86 |
|  | 50 %^b^ | 421 | 2.62 | .63 | -0.24 | 0.15 | 2.71 | 1.17 | 5.0 | 3.20 | .86 |
|  | 50% minus outliers^c^ | 412 | 2.63 | .63 | -0.26 | -0.35 | 2.71 | 1.00 | 4.20 | 3.20 | .86 |
|  | No missing in scale^d^ | 343 | 2.67 | .62 | -0.25 | -0.50 | 2.75 | 1.00 | 4.14 | 3.14 | .86 |
|  | No Missing sample^e^ | 268 | 2.56 | .63 | -0.25 | -0.50 | 2.57 | 1.00 | 4.20 | 3.14 | .86 |
|  |  |  |  |  |  |  |  |  |  |  |  |
| Context | All ^a^ | 386 | 2.65 | .65 | -0.44 | 0.13 | 2.75 | 1.29 | 5.00 | 4 | .75 |
|  | 50 %^a^ | 367 | 2.65 | .65 | 0.37 | 0.15 | 2.75 | 1.00 | 4.25 | 3.25 | .75 |
|  | 50% minus outliers^c^ | 358 | 2.64 | .64 | -0.49 | 0.17 | 2.75 | 1.00 | 4.25 | 3.25 | .75 |
|  | No missing in scale^d^ | 334 | 2.67 | .62 | -0.43 | 0.18 | 2.75 | 1.00 | 4.25 | 3.25 | .75 |
|  | No Missing sample^e^ | 268 | 2.71 | .62 | -0.43 | 0.18 | 2.75 | 1.00 | 4.25 | 3.25 | .75 |
| Patient | All ^a^ | 390 | 2.68 | .67 | 0.09 | 1.33 | 2.75 | 1.00 | 5.00 | 4 | .75 |
|  | 50 %^b^ | 366 | 2.69 | .64 | 0.02 | 1.09 | 2.71 | 1.00 | 5.00 | 3.25 | .75 |
|  | 50% minus outliers^c^ | 357 | 2.69 | .63 | -0.01 | 1.13 | 2.75 | 1.00 | 4.75 | 4.00 | .75 |
|  | No missing in scale^d^ | 310 | 2.68 | .62 | -0.11 | 0.82 | 2.75 | 1.00 | 4.75 | 3.75 | .75 |
|  | No Missing sample^e^ | 268 | 2.71 | .59 | -0.11 | 0.82 | 2.88 | 1.00 | 4.75 | 3.75 | .75 |
|  |  |  |  |  |  |  |  |  |  |  |  |
|  |  |  |  |  |  |  |  |  |  |  |  |
| BFAI Total | All ^a^ | 431 | 2.66 | .51 | 0.30 | 1.6 | 2.67 | 1.00 | 5.00 | 3.71 | .70 |
|  | 50 %^b^ | 380 | 2.61 | .45 | -0.36 | -0.31 | 2.67 | 1.48 | 3.48 | **2.11** | .67 |
|  | 50% minus outliers^c^ | 371 | 2.65 | .43 | -0.34 | -0.42 | 2.67 | 1.29 | 3.92 | 2.11 | .67 |
|  | No Missing sample^e^ | 268 | 2.65 | .41 | -0.39 | -0.36 | 2.67 | 1.48 | 3.57 | 2.29 | .67 |
|  |  |  |  |  |  |  |  |  |  |  |  |
| Note: The not applicable response option was treated as missing. M=mean, SD= standard deviation, min= minimum, Max= maximum, IQ-range= interquartile range  ^a^Scale scores with valid data in at least one item ^b^scale score with valid data for 50% of items,  ^c^Scale scores with valid data for 50% of items minus outliers. ^c^ Listwise deletion for scale, ^d^ Valid data for all items in BFAI | | | | | | | | | | | |

| **Supplemental table 5** Means, standard deviations for perceived barriers by groups of demographic and professional characteristics | | | | | | | | | |
| --- | --- | --- | --- | --- | --- | --- | --- | --- | --- |
| BFAI scale and Total | N | Innovation | Provider | Context | | Patient | | Total barrier | |
|  |  | M  (SD) | M  (SD) | M  (SD) | | M  (SD) | | M  (SD) | |
| All | 357-412 | 2.57  (.52) | 2.63  (.63) | | 2.64  (.64) | | 2.69  (.63) | | 2.65  (.43) |
| Gender |  |  |  | |  | |  | |  |
| Female | 291-341 | 2.58  (.42) | 2.64  (0.64) | | 2.66  (0.65) | | 2.69  (0.64) | | 2.66  (0.43) |
| Male | 58-64 | 2.47  (.54) | 2.52  (0.59) | | 2.53  (0.60) | | 2.69  (0.60) | | 2.58  (.41) |
| Age group |  |  |  | |  | |  | |  |
| <35 | 79-85 | 2.58  (.43) | 2.70  (.58) | | 2.69  (.68) | | 2.76  (.57) | | 2.72  (0.40) |
| 35-44 | 82-103 | 2.55  (.53) | 2.61  (.66) | | 2.63  (.72) | | 2.52  (.68) | | 2.59  (.44) |
| 45-55 | 93-111 | 2.60  (.58) | 2.61  (.63) | | 2.69  (.55) | | 2.74  (.68) | | 2.67  (.44) |
| >55 | 94-107 | 2.54  (.53) | 2.59  (.64) | | 2.57  (.61) | | 2.75  (.59) | | 2.62  (.42 |
|  |  |  |  | |  | |  | |  |
| Profession |  |  |  | |  | |  | |  |
| Nurse | 51-59 | 2.62  (.47) | 2.78  (.56) | | 2.61  (.54) | | 2.76  (0.55) | | 2.68  (0.42) |
| Counsellor | 90-96 | 2.67  (.53) | 2.75  (.59) | | 2.68  (.66) | | 2.87  (.62) | | 2.75  (0.39) |
| Psychologist | 100-108 | 2.52  (0.53) | 2.61  (0.63) | | 2.58  (0.69) | | 2.64  (.62) | | 2.63  (0.43) |
| Psychiatrist | 51-52 | 2.34  (.54) | 2.15  (0.63) | | 2.51  (.64) | | 2.38  (.63) | | 2.36  (.42) |
| Other | 58-62 | 2.61  (.47) | 2.68  (.57) | | 2.82  (.59) | | 2.73  (.64) | | 2.71  (.38) |
| Education |  |  |  | |  | |  | |  |
| Low | 9-10 | 2.52  (.52) | 2.64  (.58) | | 2.33  (.84) | | 2.64  (.61) | | 2.52  (.45) |
| Bach | 235-273 | 2.69  (.45) | 2.68  (.62) | | 2.68  (.63) | | 2.72  (.66) | | 2.68  (.43) |
| Ms | 93-107 | 2.68  (.45) | 2.56  (.63) | | 2.60  (.69) | | 2.67  (.58) | | 2.61  (.41) |
| PhD | 13-15 | 2.65  (.43) | 2.25  (.52) | | 2.63  (.44) | | 2.53  (.46) | | 2.48  (.32) |
| Tenure Child  Mental health |  |  |  | |  | |  | |  |
| <5 years | 164-179 | 2.71  (.38) | 2.70  (.58) | | 2.70  (.69) | | 2.74  (.57) | | 2.71  (.39) |
| 5-10 years | 49-60 | 2.63  (.49) | 2.56  (.68) | | 2.60  (.60) | | 2.60  (.72) | | 2.55  (.44) |
| 10-15 years | 43-48 | 2.71  (.46) | 2.54  (.69) | | 2.63  (.45) | | 2.70  (.79) | | 2.62  .44 |
| 15-20 years | 35-42 | 2.62  (.41) | 2.63  (.59) | | 2.50  (.70) | | 2.76  (.46) | | 2.59  (.40) |
| >20 years | 55-60 | 2.66  (.49) | 2.55  (.70) | | 2.61  (.64) | | 2.63  (.65) | | 2.61  (.47) |
| *Note* The non applicable response option is treated as missing. Seven multivariate outliers were excluded. Scale scores are mean scores provided that at least 50 % of items had valid data. M= mean, SD= standard deviation  *Psychiatrists include child psychiatrists, residents, and MDs without any specialist training  ** Other are auxiliary nurse and others | | | | | | | | | |

| **Supplemental table 6** Correlation matrix of the demographic characteristics and scales of the Barrier and facilitators assessment instrument (BFAI) | | | | | |
| --- | --- | --- | --- | --- | --- |
|  | n | Gender | Age | Experience | Educational level |
| Gender | 424 | - |  |  |  |
| Age | 424 | -.05 | - |  |  |
| Years of experience | 423 | -.14** | .61** | - |  |
| Education level | 425 | -.10* | -.13** | -.16* | - |
| Innovation | 382 | .04 | -.04 | -.04 | .00 |
| Provider | 412 | .07 | -.06 | -.08 | -.13** |
| Context | 358 | .08 | -.06 | -.07 | -.02 |
| Patient | 357 | .00 | .03 | -.04 | -.05 |
| BFAI Total | 371 | .06 | -.06 | -.10 | -.08 |
| Note: Gender is coded 0=male 1= female, educational level is coded 1=lower, 2=bachelor, 3= master, 4= Phd. Numbers are Pearson´s correlation coefficient., Spearman was also used with the same results.  *= p<.05, **= p<.001 (two tailed) | | | | | |

| **Supplemental table 2** Correlation matrix of the demographic characteristics and scales of the Barrier and facilitators assessment instrument (BFAI) | | | | | |
| --- | --- | --- | --- | --- | --- |
|  | n | Gender | Age | Experience | Educational level |
| Gender | 424 | - |  |  |  |
| Age | 424 | -.05 | - |  |  |
| Years of experience | 423 | -.14** | .61** | - |  |
| Education level | 425 | -.10* | -.13** | -.16* | - |
| Innovation | 382 | .04 | -.04 | -.04 | .00 |
| Provider | 412 | .07 | -.06 | -.08 | -.13** |
| Context | 358 | .08 | -.06 | -.07 | -.02 |
| Patient | 357 | .00 | .03 | -.04 | -.05 |
| BFAI Total | 371 | .06 | -.06 | -.10 | -.08 |
| Gender is coded 0=male 1= female, educational level is coded 1=lower, 2=bachelor, 3= master, 4= Phd  Numbers are Pearson´s correlation coefficient  A higher BFAI composite score indicates more barriers.  *= p<.05, **= p<.01 (two tailed) | | | | | |
